# Left atrial diameter in heart failure with left ventricular preserved, mid-range, and reduced ejection fraction

**DOI:** 10.1097/MD.0000000000018146

**Published:** 2019-11-27

**Authors:** Ning Zhu, Hao Chen, Xuyong Zhao, Fanhao Ye, Wenbing Jiang, Yi Wang

**Affiliations:** Department of Cardiology, The Wenzhou Third Clinical Institute Affiliated To Wenzhou Medical University, Wenzhou People's Hospital, Wenzhou, Zhejiang Province, China.

**Keywords:** atrial fibrillation, heart failure, left atrial diameter

## Abstract

Left atrial (LA) remodeling has been identified to predict atrial fibrillation (AF) and heart failure. However, the role of LA diameter (LAD) in patients with heart failure (HF) with preserved (HFpEF), mid-range (HFmrEF), and reduced ejection fraction (HFrEF) remains poorly understood.

A total of 142 patients including 71 subjects with AF (21 of HFpEF, 22 of HFmrEF, and 28 of HFrEF) and 71 ejection fraction (EF)-matched subjects with sinus rhythm (SR) were included in the study. Baseline characteristics and echocardiographic parameters including LAD were compared between both groups as well as among HFpEF, HFmrEF, and HFrEF.

In receiver-operating characteristic (ROC) analyses, LAD predicted AF in HFpEF, HFmrEF, and HFrEF [area under the curve (AUC): 0.646; *P* = .03]. LAD was negatively association with left ventricular ejection fraction while positively with Nt-proNP and left ventricular end-diastolic diameter (regression coefficient: −0.239, *P* = .004; regression coefficient: 0.191, *P* = .023; regression coefficient: 0.357, *P* < .001). In ROC analyses, LAD predicted HFrEF among the 3 categories (AUC: 0.629, *P* = .01).

In the setting of HF, LAD was higher in AF than in and SR, and predicted AF. Furthermore, LAD was associated with severity of HF in HFpEF, HFmrEF, and HFrEF, and also predicted HFrEF.

## 1 Introduction

The left atrium plays a key role in regulating left ventricular filling and cardiovascular performance by reserving pulmonary venous return and augmenting ventricular filling. The increasingly interest in atrial size and function has led to our better understanding of cardiovascular disease. Left atrial (LA) enlargement has been demonstrated to be a predictor of adverse cardiovascular outcomes, such as atrial fibrillation (AF), heart failure (HF), and cardiovascular death.^[1]^ It had been identified that LA dysfunction is positively correlated with reduction of exercise capacity^[2,3]^ and poor prognosis.^[4,5]^ LA remodeling and function were also compared in HF with preserved (HFpEF) and reduced Left ventricular ejection fraction (HFrEF).^[6]^ HFrEF has greater eccentric LA remodeling, whereas HFpEF has increased LA stiffness. LA function is associated with outcome more closely in HFpEF. LA diameter (LAD) is simple, convenient, and commonly used in clinical practice and research studies.^[1]^ LAD is an independent predictor of the occurrence of HF in patients with nonvalvular AF.^[7]^ LA enlargement in AF patients had a greater incidence of HF.^[8]^ To our best acknowledge, the association of LAD with HFpEF, heart failure with left ventricular mid-range (HFmrEF), and HFrEF remains unknown.

The AF is common in HF, and they share common risk factors, affect each other, and together result in a worse prognosis.^[9]^ Atrial dilatation is the major marker of left atrium remodeling and promotes the occurrence or maintenance of AF.^[10]^ Any persistent change in atrial structure or function leads to atrial remodeling. Therefore, these structural changes of LA are common in AF as a result of the high prevalence of hypertension, cardiovascular disease, and HF. The majority of data of the AF–HF relationship is based on HFrEF. Recent research showed that AF was progressively more common with increasing ejection fraction (EF), whereas associated with similar clinical characteristics in HFpEF, HFmrEF, and HFrEF.^[11]^ LA enlargement is associated with AF in the general population and ischemic stroke.^[12]^ However, whether LAD correlated with AF in HFpEF, HFmrEF, and HFrEF is poorly understood. Therefore, the aim of the present study was to investigate the relationships between LAD and HFpEF, HFmrEF, and HFrEF as well as AF.

## 2 Methods

### 2.1 Study population

This was a hospital-based single center retrospective cohort study of patients with HFpEF, HFmrEF, and HFrEF. A total of 321 consecutive subjects with HF referred to Wenzhou People's Hospital between August 2015 and March 2018 were reviewed. The patients meeting inclusion and exclusion criteria were divided to AF group and sinus rhythm (SR) group. Meanwhile, the 2 groups have the same subjects with HFpEF, HFmrEF, and HFrEF. All the patients underwent electrocardiogram. AF patients including paroxysmal and persistent were confirmed by 24-hour dynamic electrocardiogram. AF patients without ablation history were included in this study. The criterion and classification of HF were used according to the European Society of Cardiology HF guidelines. In the present study, patients with EF of ≥50% were defined as HFpEF, EF of 40% to 49% as HFmrEF, and EF of ≤39% as HFrEF. Exclusion criterion was as follows: recent acute myocardial infarction, recent stroke, recent acute coronary syndrome, chronic pulmonary heart disease, severe valvular disease, autoimmune disease, inflammatory states, and cancer. Comorbidities on admission were extracted by reviewing discharge letters. Baseline characteristics including age, gender, medical history, and medications, presenting symptoms and signs (including NYHA), a history of smoking or alcohol and laboratory values were obtained directly from the hospital information system. This study complies with the ethics review board of Wenzhou People's Hospital. Due to the retrospective nature of the study, informed consent was waived by the ethics committee. All methods were performed in accordance with the relevant guidelines and regulations. Echocardiographic examination was ensured to be conducted by an experienced echocardiographer based on the recommendations of the American Society of Echocardiography and the European Association of Cardiovascular Imaging.^[13]^ Two-dimensional and 2-dimensionally guided M-mode images were recorded from the standardized views. Left ventricular EF (LVEF), left ventricular end-diastolic diameter (LVEDd), left ventricular end-systolic diameter (LVEDs), interventricular septal thickness at end diastole (IVSd), and the left ventricular posterior wall thickness at end diastole (LVPWd) were also measured.

### 2.2 Statistical analysis

The final study population consisted of patients meeting the above mentioned criteria. SPSS 22.0 was used for the statistical analyses. All the data are presented as mean ± standard deviation. Differences in discrete variables were compared among the groups examined by using Chi-squared, the Fisher exact test, or Mann–Whitney tests. The Kruskal–Wallis tests were used of comparison of nonsymmetric continuous variables. Correlations analysis was used to determine the relationships between variables by Spearman rank correlation test. Receiver-operating characteristic (ROC) curve analysis was conducted to identify LAD predicting AF and HFrEF. *P*-values of <.05 were considered to indicate statistical significance.

## 3 Results

### 3.1 Baseline characteristics

Of 321, 142 patients met our inclusion and exclusion criteria. About 71 subjects with AF (21 of HFpEF, 22 of HFmrEF, and 28 of HFrEF) and 71 EF-matched subjects with SR were included in the study. Clinical characteristics of the 2 groups are summarized in Table 1. Age, sex distribution, smoking, and alcohol were similar between the 2 groups. Hypertension in AF group was close to SR group, while AF group had more diabetes. There were no differences in coronary heart disease, prior revascularization and dilated myocardiopathy while AF group had more prior myocardial infarction. NYHA III/IV was also similar between the 2 groups. Compared with patients with SR (43.8 ± 6.4 mm), patients with AF had the largest LAD (47.3 ± 6.1 mm). For other echocardiographic parameters including LVEF, LVEDd, LVEDs, IVSd, and LVPWd, there were no differences between the 2 groups. All the biochemical values did not differ in the 2 groups. Medical therapies were similar among the 2 groups except for warfarin (*P* < .001).

**Table 1:**
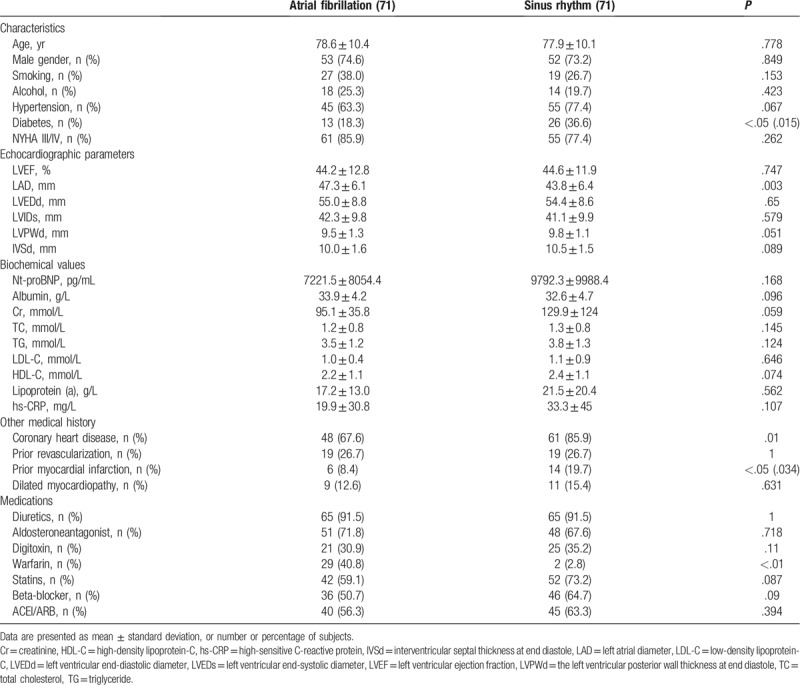
Clinical characteristics of patients according to heart rhythm.

### 3.2 ROC analysis for LAD predicting AF and association of LAD with HF

**Figure 1:**
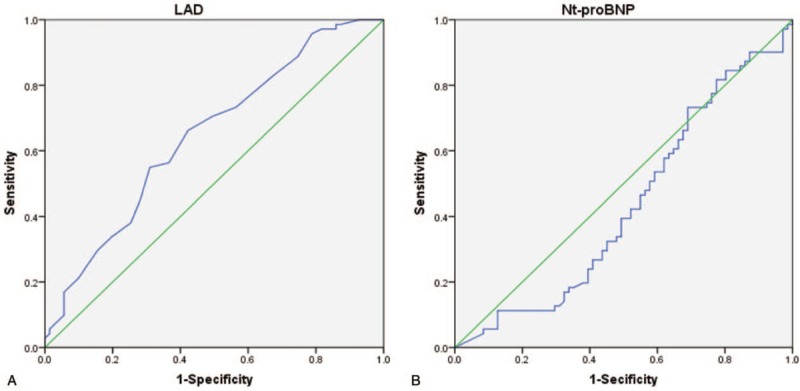
Receiver-operating characteristic analysis of (A) left atrial diameter (LAD) and (B) NT-proBNP in prediction of atrial fibrillation in heart failure.

**Figure 2:**
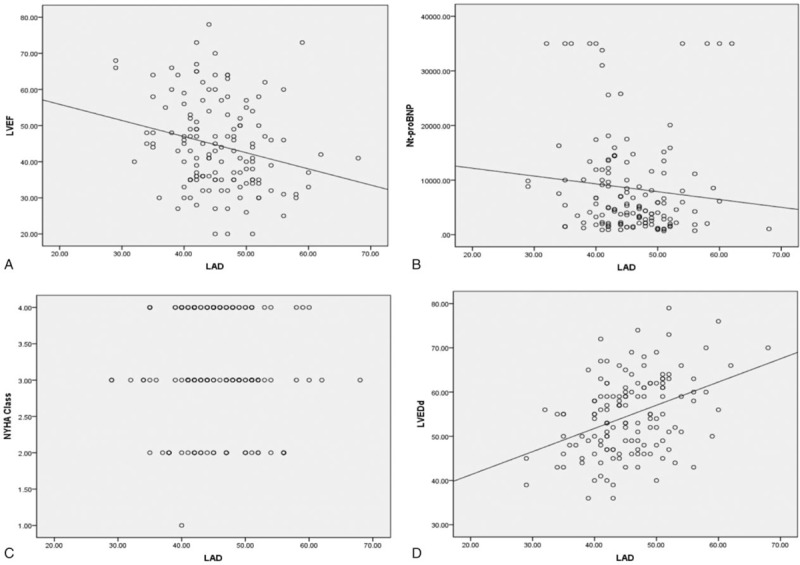
The association of left atrial diameter (LAD) with left ventricular ejection fraction (LVEF) (A), NT-proBNP (B), NYHA class (C), and left ventricular end-diastolic diameter (LVEDd) (D).

In ROC analyses, LAD predicted AF in HFpEF, HFmrEF, and HFrEF with optimal cut-off point above [area under the curve (AUC): 0.646; *P* = .03, Fig. 1], as shown in Figure 2. However, Nt-proBNP did not predict AF in HFpEF, HFmrEF, and HFrEF (AUC: 0.433; *P* = .168, Fig. 1). Figure 2 shows that LADs were negatively associated with LVEF while positively with Nt-proNP and LVEDd (regression coefficient: −0.239, *P* = .004; regression coefficient: −0.191, *P* = .023; regression coefficient: 0.357, *P* < .001). Data also showed there was no correlation of LAD with NYHA Class (regression coefficient: 0.054, *P* = .521).

### 3.3 Relationship between LAD and other variables

For other risks for HF, LAD were positively with coronary heart disease, prior myocardial infarction and hypertension, but not diabetes (regression coefficient: 0.2, *P* = .017; regression coefficient: 0.225, *P* = .007, regression coefficient: 0.178, *P* = .034; regression coefficient: 0.15, *P* = .076, Table 2).

**Table 2:**
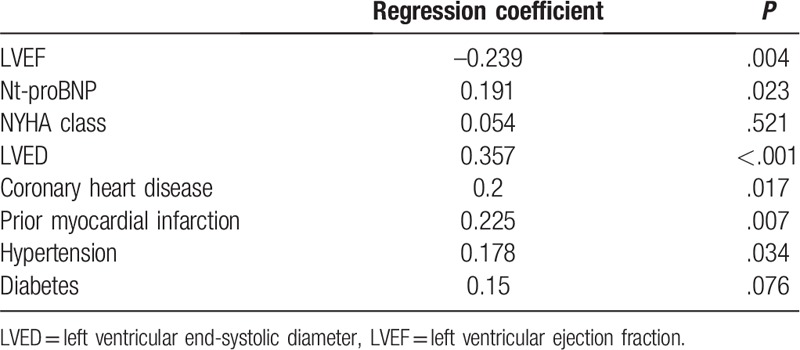
Association of LAD with variables.

### 3.4 LAD in HFpEF, HFmrEF, and HFrEF and ROC analysis based on LVEF

**Figure 3:**
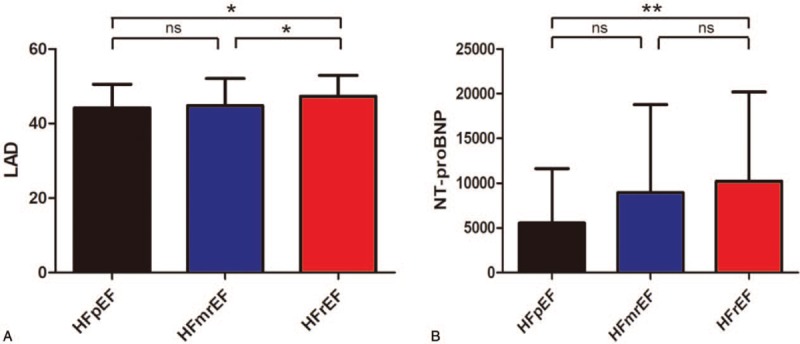
Left atrial diameter (LAD) and Nt-preBNP in HFpEF, HFmrEF, and HFrEF. ^∗^*P* < .05, ^∗∗^*P* < .01.

**Figure 4:**
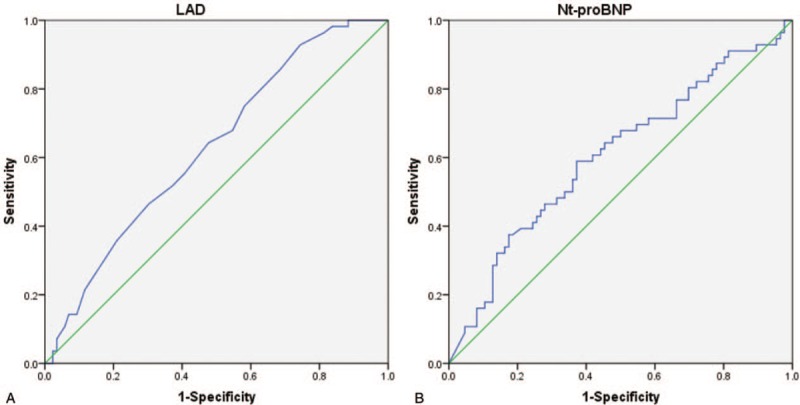
Receiver-operating characteristic analysis of (A) left atrial diameter (LAD) and (B) NT-proBNP in prediction of HFrEF among the 3 categories.

To determine LAD in the 3 categories and the potential prediction of LAD for HFrEF, we divided the subjects into 3 groups based on LVEF. As were showed in Figure 3, HFrEF had greater LAD than HFpEF and HFmrEF (47.3 ± 5.6 vs 44.1 ± 6.3, *P* = .026; 47.3 ± 5.6 vs 44.7 ± 7.3, *P* = .031), but LAD in HFpEF and HFmrEF was similar (44.1 ± 6.3 vs 44.7 ± 7.3, *P* = .983). We also found Nt-proBNP in HFrEF was higher than HFpEF (10,241.2 ± 9954.3 vs 5560.7 ± 6054.2, *P* = .007); however, there were no differences between HFpEF and HFmrEF, as well as HFmrEF and HFpEF (5560.7 ± 6054.2 vs 8941.7 ± 9831.8, *P* = .090; 8941.7 ± 9831.8 vs 10,241.2 ± 9954.3, *P* = .331). ROC analysis showed LAD and NT-proBNP predicted HFrEF among the 3 categories (AUC: 0.629, *P* = .01; AUC: 0.607, *P* = .032, Fig. 4).

## 4 Discussion

To identify diagnostic utility of LAD for AF in HFpEF, HFmrEF, and HFrEF, 71 subjects with AF and 71 EF-matched subjects with SR were included in the study. In this study, we found the prediction of LAD, but not Nt-proBNP, for AF in HFpEF, HFmrEF, and HFrEF. The results indicated that LAD was associated with LVEF, Nt-proBNP, and LVEDd expect for NYHA class. Furthermore, LAD was also correlated with coronary heart disease and hypertension. After all the subjects were divided according to LVEF, data showed that LAD in HFrEF was greater than HFpEF and HFmrEF, but HFpEF is close to HFmrEF. HFrEF has higher Nt-proBNP than HFpEF and HFmrEF while HFmrEF is similar to HFpEF and HFrEF. Finally, LAD predicted HFrEF among the 3 categories.

Atrial dilatation is the major marker of left atrium remodeling. LA enlargement could predict the development of 1st AF.^[14]^ Furthermore, it has been showed that patients with incident chronic HF during follow-up had greater LAD.^[15]^ Due to atrial structural remodeling, mainly because of fibrosis, AF patients always had greater LAD.^[16]^ Indeed, the finding of the present study indicated LAD in AF was still higher than SR in the setting of HF. Although it has been reported that HF patients were associated with larger LA dilatation than HF-free controls and LA remodeling and function differed in HFpEF (LVEF ≥ 50%) and HFrEF (LVEF < 50%).^[6]^ To eliminate the effects of LVEF, AF patients and SR patients enrolled in this study were EF matched. Our ROC analysis also showed LAD could predict AF in HF.

The association of LA enlargement with HF has been well established. LA was accompanied remodeling, apoptosis, myosin isoform expression, collagen matrix turnover, and reduced intrinsic contractility when response to increased loading. In SOLVD Registry and Trials, LAD was associated with increased risk of death and cardiovascular hospitalization.^[17]^ LA area is also a powerful predictor of death or hospitalization among HF patients with predominantly impaired systolic function.^[18]^ Furthermore, LA volume index predicted chronic HF hospitalization and mortality as well as LVEF in patients with coronary disease.^[19]^ 3D LAV >100 mL predicted cardiac deaths and hospitalizations as a result of heart failure among patients with severe LV dysfunction.^[20]^ In the present study, we also found LAD was associated with LVEF. There was a significant correlation with LAD index and left ventricular filling pressure; therefore, LAD directly reflected the left ventricular filling pressure.^[5]^ As was shown in this study, although LAD was not associated with NYHA class because of the subjective assessment factors, LAD was associated with Nt-proBNP and LVEDd.

Several risk factors, such as hypertension, diabetes mellitus, and coronary artery disease contributed to development of HF. LA appendage stores about 30% of ANP, which regulates natriuresis and diuresis.^[21]^ Although ANP levels did not have impact on systemic blood pressure, a recent study showed that after LA appendage exclusion, systemic blood pressure was reduced in patients with AF and history of hypertension.^[22]^ Furthermore, the indexed LAD correlated positively with diabetes mellitus, hypertension, and coronary artery disease.^[1]^ In fact, high blood pressure induced anatomic and hemodynamic changes, which was associated with atrial wall stress, and decreasing left ventricular diastolic pressure, subsequently affecting clinical outcomes in HF. Our data showed LAD was also associated with hypertension. Patients with AMIs followed for a mean of 15 months, higher LA volume index was a powerful predictor of all-cause mortality.^[23]^ AMIs and larger LA volume index was correlated with a higher incidence of chronic HF, increased LV dimensions, and reduced LVEF.^[24]^ The result showed LAD was correlated with coronary heart disease and prior myocardial infarction. In this study, LAD was not associated with diabetes, although diabetes mellitus was independent correlates of LA fibrosis and poor outcomes.^[25,26]^

In several studies, LA remodeling has been compared in HFpEF and HFrEF. LA maximal volume and active emptying fraction were greater in systolic HF (LVEF < 0.5) than diastolic HF (LVEF ≥ 0.5).^[27]^ Moreover, HFrEF (LVEF < 0.5) patients had larger LA volumes than HFpEF (LVEF ≥ 0.5).^[6]^ There was no difference in LV mass and LA volume between diastolic HF and systolic HF, but the sample size of this study was very small.^[28]^ In the present study, LAD increased accompanying with decreased LVEF. And LAD predicted HFrEF across the 3 categories.

Although LAD may less precisely represent the true LA size than LA volume, LAD is still a reliable surrogate. Furthermore, measurement of LAD is more easily conducted than LA volume and has already been included in the routine echocardiographic examination. Despite not wide application, several study also reported that LAD could be used to predict clinical outcomes.^[18,29]^

In conclusion, in the setting of HF, LAD was higher in AF than in and SR and predicted AF. Furthermore, LAD was associated with severity of HF, and also predicted HFrEF across the three categories. However, it is hard to permit any definite conclusions due to the small sample. Prospective studies with long-term follow-up are required to evaluate the role of LAD in HFpEF, HFmrEF, and HFrEF.

### 4.1 Limitation

The present study has some limitations. The sample size was small. Furthermore, our study was observational study and longitudinal data are necessary to evaluate the role of LAD in HF. Finally, follow-up study should be conducted to elucidate the predictive role of LAD for outcome of HF. All the patients with paroxysmal could not be confirmed by 24-hour dynamic electrocardiogram.

## Acknowledgment

The authors thank all the study personnel for their contribution.

## Author contributions

**Data curation:** Hao Chen, Xuyong Zhao, Fanhao Ye.

**Formal analysis:** Wenbing Jiang, Yi Wang.

**Supervision:** Ning Zhu.

**Writing – original draft:** Wenbing Jiang.

**Writing – review & editing:** Ning Zhu.

Ning Zhu orcid: 0000-0002-7521-6266.
